# Personalisierte Sepsistherapie und moderne Studiendesigns

**DOI:** 10.1007/s00063-026-01495-6

**Published:** 2026-09-15

**Authors:** Sascha David, Rolf Erlebach, Alix Buhlmann, Mattia Müller

**Affiliations:** 1 Institut für Intensivmedizin, Universitätsspital Zürich, Rämistrasse 100, 8032 Zürich, Schweiz; 2 Medizinische Fakultät, Universität Zürich, Zürich, Schweiz; 3 Nieren- und Hochdruckerkrankungen, Medizinische Hochschule Hannover, Hannover, Deutschland

**Keywords:** Phänotypische Variabilität, Endpunktbestimmung, Hämadsorption, Präzisionsmedizin, Immuntherapie, Phenotypic variability, Endpoint determination, Hemadsorption, Precision medicine, Immunotherapy

## Abstract

Bei der Sepsis handelt es sich um eine lebensbedrohliche Organdysfunktion als Folge einer Infektion. Trotz jahrzehntelanger Forschung existiert bis heute kaum Evidenz für kausale Therapieansätze gegen diese Wirtsantwort. Die Diskrepanz zwischen stetig wachsendem biologischen Verständnis und begrenztem klinischen Fortschritt wird häufig als „translational gap“ bezeichnet. Randomisierte Studiendesigns waren zum einen auf heterogene Patientenkollektive fokussiert und nutzten zum anderen die Mortalität als primären Endpunkt. Dadurch besteht die Gefahr, dass potenziell wirksame Interventionen in biologisch unterschiedlichen Patientengruppen verwässert werden und potenziell relevante Effekte statistisch nicht erkennbar sind. Die Fülle an negativen Sepsisstudien hat nicht nur zu einer Art Fatigue der Wissenschaftler, sondern auch zu einer Angst vor finanziellem Schaden für die forschende Industrie geführt. Neuere Studien deuten jedoch darauf hin, dass präzisionsmedizinische Ansätze einen Ausweg aus dieser Situation bieten könnten. Die aktuellen Untersuchungen ANDROMEDA-SHOCK‑2, ImmunoSep und Tigris nutzen physiologische oder immunologische Phänotypen zur Patientenselektion und kombinieren diese mit innovativen Endpunkten und modernen statistischen Methoden. Gemeinsam zeigen sie, dass eine stärkere Ausrichtung an der zugrunde liegenden Pathophysiologie klinisch relevante Vorteile sichtbar machen kann, die in klassischen Studiendesigns verborgen bleiben. Diese Entwicklungen markieren einen grundlegenden Wandel in der Sepsisforschung: weg von universellen Therapiekonzepten, hin zu individualisierten, biologisch begründeten und adaptiven Behandlungsstrategien.

## Abstract (en)

Sepsis is a life-threatening organ dysfunction due to a pathological host response to infection. Despite decades of research, there is still very limited evidence for causal therapeutic approaches targeting this injurious host response. The discrepancy between steadily increasing biological understanding and limited clinical progress is often referred to as the “translational gap.” On the one hand, randomized trial designs have focused on heterogeneous patient populations, and on the other, they have mostly used mortality as the primary endpoint. As a result, there is a risk that potentially effective interventions are diluted across biologically diverse patient groups, making relevant effects statistically undetectable. The large number of negative sepsis trials has not only led to fatigue among researchers, but also to concerns about financial risks within the research-driven pharmaceutical industry. More recent studies, however, suggest that precision medicine approaches could offer a way out of this situation. Recent investigations such as ANDROMEDA SHOCK 2, ImmunoSep, and Tigris use physiological or immunological phenotypes for patient selection and combine these with innovative endpoints and modern statistical methods. Together, they demonstrate that a stronger focus on the underlying pathophysiology can reveal clinically relevant benefits that remain hidden in conventional trial designs. These developments mark a fundamental shift in sepsis research: away from universal treatment concepts for everybody and toward individualized, biologically grounded, and adaptive therapeutic strategies.

Sepsis ist eine lebensbedrohliche Organdysfunktion als Folge einer pathologischen Wirtsreaktion auf eine Infektion [1] mit einer sehr hohen Mortalität. Trotz 30-jähriger Bemühungen in randomisierten Studien wurden keine relevanten Fortschritte in der kausalen Behandlung erzielt. Die Einführung einer frühen Antibiotikagabe, von Algorithmen zur hämodynamischen Stabilisierung und generell klar strukturierten Behandlungspfaden hat die Prognose vieler Betroffener zwar etwas verbessert, doch sterben etwa 40 % aller PatientInnen mit septischem Schock [2]. Fehlende spezifische Therapieoptionen, die gezielt in die krankheitsauslösenden Mechanismen eingreifen [3, 4] haben bei KlinikerInnen, WissenschaftlerInnen und der forschenden Industrie zu erheblicher Frustration geführt.

Zahlreiche Wirkstoffe mit an sich überzeugenden präklinischen Daten scheiterten später in randomisierten klinischen Studien (RCTs). Daraus entstand teilweise die Auffassung, Sepsis sei grundsätzlich nicht für einen gezielten Therapieansatz zugänglich. Eine solche Interpretation greift jedoch zu kurz. Die Ursache liegt möglicherweise weniger im Fehlen wirksamer Medikamente als vielmehr darin, dass versucht wurde, biologisch unterschiedliche Erkrankungen wie eine einzige Krankheit zu behandeln. Interessanterweise deuten genau darauf die positiven Ergebnisse dreier Sepsis-RCTs der vergangenen Monate hin.

Die Zukunft der Sepsistherapie liegt zunächst in der richtigen PatientInnenselektion

Der vorliegende Beitrag diskutiert daher nicht alle Sepsisstudien der letzten Jahrzehnte, sondern konzentriert sich auf die Frage, warum so viele Studien erfolglos blieben und welche neuen Konzepte einen Weg nach vorn eröffnen könnten. Im Mittelpunkt steht die Hypothese, dass die biologische Heterogenität der Erkrankung bislang nicht ausreichend berücksichtigt wurde, also die fehlende Individualisierung einer gezielten Therapie*. *Somit liegt die Zukunft der Sepsistherapie nicht primär in neuen Medikamenten, sondern zunächst in der richtigen PatientInnenselektion.

## Warum so viele Sepsisstudien scheiterten

Ein zentrales Problem besteht in der außerordentlichen klinischen und biologischen Heterogenität des Sepsissyndroms [1]. Hinter dem gemeinsamen klinischen Begriff verbergen sich sehr unterschiedliche Krankheitsbilder. PatientInnen unterscheiden sich hinsichtlich Infektionsquelle, Erregerspektrum, genetischer Prädisposition, Vorerkrankungen, Alter, Immunstatus und Organreserve [5]. Traditionelle Studien wenden oft standardisierte Protokolle auf eine heterogene Sepsispopulation an. Dieser Ansatz kann zwangsläufig keinen Nettoeffekt zeigen [6]. Starre Einschlusskriterien und traditionelle statistische Konzepte erschweren die Identifikation relevanter Effekte. Viele Studien wurden unter der Annahme geplant, dass ein einzelner Therapieansatz bei nahezu allen PatientInnen mit Sepsis gleich wirkungsvoll ist. Heute erscheint diese Vorstellung zunehmend unrealistisch. Gleichzeitig sinkt das Interesse der Pharmaindustrie, wenn die Einengung der Zielgruppe zu schmal und damit der potenzielle Absatzmarkt zu klein wird.

Besonders bedeutsam ist die Dynamik der Immunantwort. Während einige PatientInnen eine ausgeprägte Hyperinflammation entwickeln, zeigen andere schon früh Zeichen einer gewissen Immunsuppression [5, 7]. Häufig existieren beide Zustände gleichzeitig oder wechseln sich im Krankheitsverlauf ab. Die Zuordnung einzelner PatientInnen einer großen Population zum einen oder anderen Phänotyp ist im Alltag sehr schwierig und es besteht ein hohes Risiko einer Vermischung biologisch unterschiedlicher Populationen. Die SENECA-Studie konnte kürzlich mithilfe maschinellen Lernens 4 Sepsisphänotypen identifizieren, die sich hinsichtlich klinischer Merkmale, Biomarkerprofile und Sterblichkeitsraten (5–40 %) drastisch unterscheiden [8]. Simulationen im Rahmen des Projekts verdeutlichten das Problem der „Verwässerung“ von Effekten: Das Ergebnis einer Studie kann allein durch eine veränderte Verteilung dieser Phänotypen in der Studienpopulation von einem relevanten Nutzen zu einem signifikanten Schaden umschlagen.

Besonders bedeutsam ist die Dynamik der Immunantwort

Ein weiteres Problem betrifft die Wahl des primären Endpunkts. Seit Jahrzehnten fokussierten wir uns v. a. darauf, nachzuweisen, dass eine spezifische Therapie das Überleben der PatientInnen verbessert. Die 28- oder 90-Tage-Mortalität wird allerdings von zahlreichen Faktoren, die nur teilweise mit der untersuchten Intervention zusammenhängen, beeinflusst. Frailty, Vorerkrankungen, Therapieziele, Rehabilitationsmöglichkeiten und persönliche Entscheidungen zur Therapiebegrenzung wirken sich erheblich auf das Überleben aus. Selbst eine biologisch wirksame Therapie kann daher in einer Mortalitätsanalyse unsichtbar bleiben. Diese Überlegungen wurden in Tab. 1 zusammengefasst und teilweise ergänzt.

**Tab. 1:** Gründe für das Scheitern von Sepsisstudien

Problemfeld	Beschreibung	Konsequenz
Klinische Heterogenität	Unterschiedliche Infektionsquellen, Erreger, Komorbiditäten, Alter, Immunstatus und Organreserve	Wirksame Effekte durch heterogene Populationen verwässert
Biologische Heterogenität	Unterschiedliche molekulare Mechanismen und Immunantworten innerhalb des Sepsissyndroms	Nur Subgruppen könnten profitieren
Dynamik der Immunantwort	Hyperinflammation und Immunsuppression können gleichzeitig oder zeitlich versetzt auftreten	Falsches Timing der Intervention reduziert den Therapieeffekt
Starre Ein- und Ausschlusskriterien	Traditionelle Studiendesigns berücksichtigen individuelle Unterschiede unzureichend	Geringere Chance, relevante Effekte zu erkennen
Ungeeignete Endpunkte	Fokus auf 28- oder 90-Tage-Mortalität	Biologische Effekte bleiben möglicherweise unsichtbar
Einfluss konkurrierender Faktoren	Frailty, Komorbiditäten, Therapielimitationen und Rehabilitation beeinflussen das Überleben	Mortalität spiegelt Interventionseffekt nur unzureichend wider
Ökonomische Hürden	Kleine Zielgruppen durch Individualisierungsansätze reduzieren das Interesse der Industrie	Weniger Investitionen in präzisionsmedizinische Ansätze

## Präzisionsmedizin

Die Präzisionsmedizin verfolgt das Ziel, PatientInnengruppen anhand biologischer, immunologischer oder physiologischer Merkmale zu identifizieren und gezielt zu behandeln [1]. Man spricht in diesem Zusammenhang auch von einer „individualisierten Therapie“. Statt alle Betroffenen gleich zu therapieren, sollen diejenigen ausgewählt werden, bei denen ein bestimmter Mechanismus tatsächlich krankheitsrelevant ist. Dies klingt kompliziert, ist aber an einem einfachen Beispiel aus der kardiovaskulären Medizin sehr gut nachzuvollziehen. Ein erhöhtes LDL-Cholesterin ist pathophysiologisch mit der Entwicklung einer koronaren Herzerkrankung assoziiert; gleichzeitig kann die Biosynthese von Cholesterin mittels Inhibition der HMG-CoA-Reduktase (also mit Statinen) spezifisch gehemmt werden. Analog dieser Kausalkette lässt sich postulieren, dass wir auch bei der Sepsis zuerst pathophysiologisch relevante Targets identifizieren müssen, um dann in einem spezifischen Ansatz eine therapeutische Beeinflussung dieses Pfads in Studien zu untersuchen.

Bekannterweise hat dieses Konzept in der Onkologie bereits zu erheblichen Fortschritten geführt (Beispiel hormonrezeptorpositive Karzinome). Auch bei der Sepsis gewinnt die Idee an Bedeutung. Moderne Biomarker, molekulare Analysen und digitale Auswertungsverfahren ermöglichen zunehmend die Identifikation von Subgruppen mit spezifischen Risikoprofilen und möglicherweise auch therapeutischen Angriffspunkten [7, 9]. Die jüngsten Sepsisstudien liefern wichtige Hinweise darauf, dass dieser Ansatz auch in der Intensivmedizin praktikabel sein könnte.

## Drei Hoffnungsträger 2025/26

### Die ImmunoSep-Studie

**Abb. 1:**
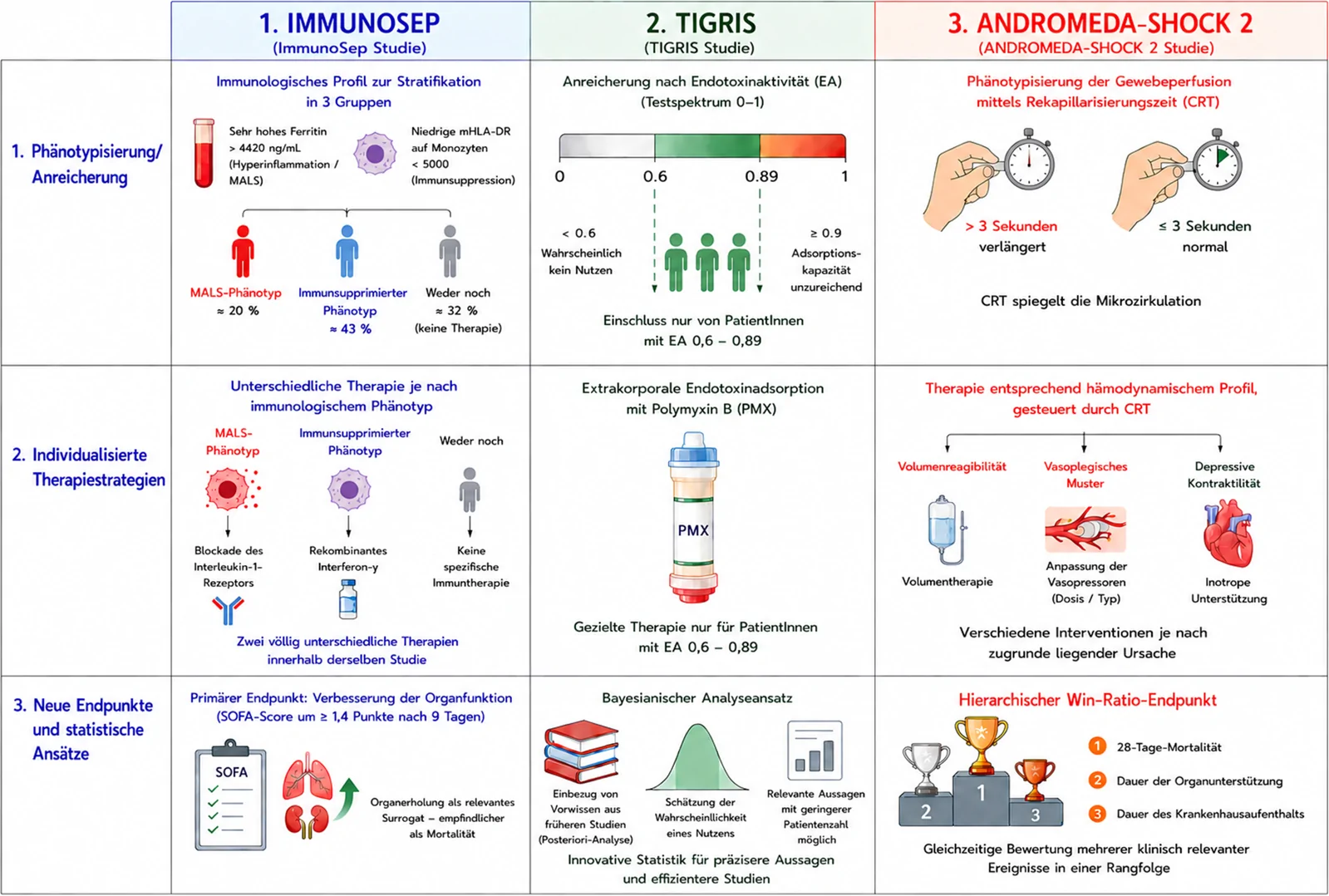
Darstellung der Kerninhalte dieser Arbeit: Stratifizierung, Therapieindividualisierung und neue Endpunkte bzw. statistische Ansätze. (Die Abbildung wurde mithilfe des KI-Tools Open AI/ChatGPT‑5.5 Instant erstellt.)

Die ImmunoSep-Studie stellt einen paradigmatischen Schritt in Richtung immunologische Präzisionsmedizin dar ([10]; Tab. 2). Die PatientInnen wurden anhand einfacher immunologischer Profile, basierend auf vorherigen Pilotstudien, in 3 Gruppen stratifiziert [4]. Ein sehr hohes Ferritin (> 4420 ng/ml) wurde als Zeichen einer dominierenden Hyperinflammation, einer Art Makrophagenaktivierungssyndrom (MALS), gewertet und eine Reduktion der Expression an HLA-DR auf Monozyten (mHLA-DR < 5000) als Zeichen der Immundepression (Abb. 1). In der Pilotstudie konnten anhand dieser beiden Parameter 42,9 % der PatientInnen einem immunsupprimierten Phänotyp, 20 % dem MALS-Phänotyp und 31,7 % keinem der beiden Phänotypen zugeordnet werden. Nur bei den inflammierten PatientInnen mit MALS wurde eine Blockade des Interleukin-1-Rezeptors vs. Placebo evaluiert, wohingegen PatientInnen mit einer Immunparalyse mit rekombinantem Interferon‑γ vs. Placebo behandelt wurden. Die Zwischengruppe, die nicht klar zugeordnet werden konnte, erhielt keine Therapie. Somit kamen zwei völlig unterschiedliche Therapien innerhalb derselben Studie zum Einsatz.

Als primärer Endpunkt einer personalisierten Immuntherapie nach diesem Vorgehen wurde die Verbesserung der Organfunktion anhand des Sequential Organ Failure Assessment (SOFA-)Scores gewählt (mindestens 1,4 Punkte nach 9 Tagen). Diese Entscheidung ist von besonderer Bedeutung, da der tägliche SOFA-Score die Dynamik der Organerholung abbilden kann. Tatsächlich zeigte die Studie, dass 35,1 % der PatientInnen der Behandlungsgruppe eine entsprechende SOFA-Verbesserung aufwies, aber nur 17,9 % der Kontrollgruppe. Wäre jedoch ausschließlich die Mortalität betrachtet worden, wäre dieses Signal übersehen worden. ImmunoSep demonstriert daher eindrucksvoll den Nutzen biologisch fundierter PatientInnenselektion und klinisch relevanter Surrogat-Endpunkte.

Die Inspire-Studie der gleichen Studiengruppe [11] nutzte einen ähnlichen Ansatz und sei daher kurz erwähnt. Sie untersuchte PatientInnen mit Pneumonie und erhöhtem Risiko für die Entwicklung einer Sepsis. Grundlage der Selektion waren erhöhte Presepsinspiegel. Statt bereits etablierte schwere Organversagen zu behandeln, zielte die Studie auf die Verhinderung der Krankheitsprogression ab. Dadurch wurde ein präventiver Ansatz verfolgt, der die Übergangsphase zwischen Infektion und manifester Sepsis adressiert. Trotz sehr geringer PatientInnenzahl zeigte sich ein relevanter Vorteil zugunsten der Behandlung.

### Die Tigris-Studie

Die Tigris-Studie untersuchte die extrakorporale Endotoxinadsorption mittels Polymyxin‑B (PMX) bei PatientInnen mit septischem Schock und einem SOFA-Score > 11 ([12]; Tab. 2). Der entscheidende Aspekt, der hier unterstrichen werden soll, liegt weniger in der Therapie selbst als im Studiendesign. Die KollegInnen nutzten nämlich eine Messung der Endotoxinaktivität (EA) (Testspektrum 0 bis 1) als selektives Einschlusskriterium (Abb. 1). Der Grundgedanke ist einfach: Logischerweise können nur Patienten mit erhöhter EA auch von einer Endotoxinadsorption profitieren. Basierend auf einer Post-hoc-Analyse der EUPHRATES-Studie [13] wurden sogar nur solche Patienten ausgewählt, deren EA in einem bestimmten Bereich lag (0,6–0,89; [14]), da hier die Wahrscheinlichkeit eines Nutzens am höchsten erschien. Zugrunde liegt der Gedanke, dass PatientInnen auf der niedrigen Seite des EA-Spektrums (< 0,6) keine adjuvante Endotoxinadsorption benötigen, um eine Besserung zu erzielen, wohingegen PatientInnen auf der anderen Seite des EA-Spektrums (≥ 0,9) so viel Endotoxin im Blut haben, dass die Adsorptionskapazität nicht ausreichen würde und es somit auch keinen klinischen Effekt erzeugen könnte. In diesem Zusammenhang sollte beachtet werden, dass diese Grenzen nicht vorher festgelegt waren, sondern sich aus der angesprochenen Post-hoc-Analyse ergaben [14].

Neben dem Aspekt der individualisierten PatientInnenselektion wurde außerdem ein Bayes-Analyseansatz verwendet. Im Gegensatz zu klassischen frequentistischen Verfahren integriert die Bayes-Statistik vorhandenes Wissen explizit in die Analyse, sodass frühere Studienergebnisse der Post-hoc Analyse als Vorinformationen in die Berechnung einfließen [15]. Dadurch können klinisch relevante Aussagen mit geringeren Fallzahlen erreicht werden. Gleichzeitig entsteht ein differenzierteres Verständnis der Wahrscheinlichkeit eines Behandlungserfolgs. Dieser Ansatz könnte insbesondere in der Sepsisforschung von Nutzen sein, da große, kostenintensive Studien zunehmend schwieriger durchzuführen sind.

Die 2026 publizierte Tigris-Studie zeigte ein um 6 % besseres 28-Tage- und ein um 17 % besseres 90-Tage-Überleben [12]. Diese Ergebnisse wurden auf wissenschaftlichen Kongressen viel diskutiert. Ein Kritikpunkt lag in der hohen Zahl gescreenter PatientInnen (14.890), um gerade einmal 157 Patienten einzuschließen. Aus unserer Sicht könnte aber genau dieser Punkt eine Stärke darstellen und den Präzisionsansatz unterstreichen. Eine Endotoxinadsorption ist eben keine Therapie für alle 50 Mio. Sepsisfälle, sondern stellt einen hochspezifischen Ansatz für gewisse PatientInnen mit septischem Schock, einem hohen SOFA-Score und einer deutlich, aber nicht massiv erhöhten EA zwischen 0,6 und 0,89 dar.

### Die ANDROMEDA-SHOCK-2-Studie

Die internationale multizentrische Studie ANDROMEDA-SHOCK‑2 untersuchte an ca. 1500 PatientInnen eine personalisierte hämodynamische Stabilisierungsstrategie bei septischem Schock ([16]; Tab. 2). Im Zentrum stand die sog. Rekapillarisierungszeit („capillary refill time“, CRT) als einfacher Marker der Gewebeperfusion (Abb. 1). Anstatt ausschließlich klassische makrohämodynamische Parameter wie Blutdruck oder zentralvenösen Druck zu betrachten, richtete sich die Therapie an der tatsächlichen Gewebeversorgung aus. Je nach zugrunde liegendem hämodynamischen Profil (besonders beeinflusst durch Pulsdruckamplitude und diastolischen Blutdruck) erfolgten unterschiedliche Maßnahmen, z. B Volumentherapie, Vasopressoranpassungen oder inotrope Unterstützung. Dieses Konzept berücksichtigt, dass identische Blutdruckwerte bei unterschiedlichen PatientInnen völlig verschiedene Ursachen haben können. Die CRT ist zentraler Bestandteil dieses Algorithmus und ermöglicht dabei eine einfache, kostengünstige und unmittelbar verfügbare Beurteilung der Mikrozirkulation.

Auch die benutze Statistik ist in gewisser Weise bemerkenswert, da die KollegInnen aus Chile einen hierarchischen „Win-ratio-Endpunkt“ gewählt hatten [17]. Dadurch konnten mehrere klinisch relevante Ereignisse (Mortalität, Dauer des vitalen Supports und Dauer des Krankenhausaufenthalts) gleichzeitig bewertet werden.

Es gab 131.131 „wins“ (48,9 %) in der CRT-Gruppe gegenüber 112.787 „wins“ (42,1 %) in der Standardtherapiegruppe hinsichtlich des hierarchisch zusammengesetzten primären Endpunkts. Dies entsprach einer „win ratio“ von 1,16 (1,02–1,33; *p* = 0,04).

Zusammengefasst zeigt die Studie, dass bei PatientInnen mit frühem septischen Schock eine individualisierte hämodynamische Stabilisierungsstrategie mit Steuerung anhand der CRT zu einer Verbesserung des primären kombinierten Endpunkts, hauptsächlich bedingt durch eine kürzere Dauer der Organunterstützung, führt.

**Tab. 2:** Besonderheiten der vorgestellten aktuellen Studien.

Aspekt	ImmunoSep	Tigris	ANDROMEDA SHOCK 2
*Therapeutisches Ziel*	Immunmodulation	Endotoxin-Elimination	Personalisierte hämodynamische Stabilisierung
*Konzept*	Unterschiedliche immunologische Endotypen (Hyperinflammation vs. Immunsuppression)	Endotoxin als relevanter Treiber des septischen Schocks	Mikrozirkulationsstörung und inadäquate Gewebeperfusion
*Selektionskriterien*	Ferritin > 4420 ng/ml (MALS) bzw. mHLA-DR < 5000 (Immunparalyse)	Endotoxinaktivität (EA 0,6–0,89) und SOFA > 11	CRT, Pulsdruckamplitude und diastolischer Blutdruck
*Therapie*	Anakinra bei MALS, Interferon‑γ bei Immunparalyse	Polymyxin-B-Hämoperfusion	Individualisierte Volumen‑, Vasopressor- und Inotropikatherapie
*Primärer Endpunkt*	SOFA-Verbesserung nach 9 Tagen	28- und 90-Tage-Mortalität	Hierarchischer „Win-ratio-Endpunkt“
*Besonderheit Statistik*	Fokus auf Surrogat-Endpunkte	Bayes-Statistik mit Vorwissen	Hierarchische „Win-ratio-Analyse“, Kombination aus Mortalität, Dauer der Organunterstützung u.- Krankenhausaufenthalt
*Wichtigstes Ergebnis*	Signifikante Verbesserung der Organfunktion	6 % besseres 28-Tage- und 17 % besseres 90-Tage-Überleben	„Win ratio“ 1,16 zugunsten der Interventionsgruppe
*Stärke*	Konsequente immunologische Präzisionsmedizin	Extrem selektive biomarkerbasierte Rekrutierung	Individualisierte Therapie anhand der Mikrozirkulation
*Limitationen*	Komplexe Immunphänotypisierung im Alltag	Sehr hohe Screening-zu-Einschluss-Rate (14.890: 157)	Algorithmus und CRT-Messung benötigen Schulung

*CRT *Rekapillarisierungszeit,* MALS „*macrophage activation-like syndrome“, *SOFA* Sequential Organ Failure Assessment Score

## Neue Endpunkte

Obwohl die Sterblichkeit für den Kliniker den schwerwiegendsten Endpunkt darstellt, verdeutlichen die diskutierten Studien die Grenzen klassischer Mortalitätsendpunkte. Organfunktionsverläufe, Beatmungs- oder vasopressorfreie Tage, Lebensqualität, funktionelle Erholung und zusammengesetzte hierarchische Endpunkte können zusätzliche Informationen liefern. Insbesondere der „Win-ratio-Ansatz“ erlaubt eine Priorisierung klinisch bedeutsamer Ereignisse. Während die Mortalität weiterhin am höchsten gewichtet wird, können dennoch andere relevante Behandlungseffekte berücksichtig werden. Dadurch entsteht ein umfassenderes Bild des Therapieeffekts. Die Interpretation derartiger Endpunkte ist jedoch nicht trivial. Ein statistisch signifikantes Ergebnis bedeutet nicht automatisch einen klinisch relevanten Nutzen. Moderne Endpunkte sollten daher stets zusammen mit klassischen Effektmaßen beschrieben und auch bewertet werden. Zu den zunehmend relevanten Endpunkten zählen außerdem „days alive and out of hospital“, „major adverse kidney events“, patientenberichtete Endpunkte, Langzeitkognition, Frailty und die gesundheitsbezogene Lebensqualität. Diese Parameter erweitern die klassische Mortalitätsbetrachtung um Aspekte der funktionellen und patientenzentrierten Erholung.

## Bedeutung für die zukünftige Forschung

Die beschriebenen Entwicklungen bedeuten nicht, dass etablierte Leitlinien aufgegeben werden sollten. Alle drei vorgestellten Studien sind kurz nach der Konsertierungsphase der Surviving Sepsis Campaign Guideline 2026 erschienen und konnten daher nicht mehr in deren Empfehlungen berücksichtigt werden. Eine schwierige Frage zielt in Richtung der harmonischen Kombination aus Standardisierung und Individualisierung. Zukünftige Behandlungsprotokolle könnten feste Entscheidungsstrukturen mit biologischen Markern und physiologischen Parametern verknüpfen. Dadurch bliebe die Versorgung standardisiert und reproduzierbar, während gleichzeitig patientenspezifische Besonderheiten berücksichtigt werden. Datenbasierte Entscheidungshilfen könnten diesen Prozess in Zukunft gezielt unterstützen. Für uns IntensivmedizinerInnen bedeutet dies einen gewissen Wandel der Denkweise. Die Frage lautet künftig weniger, welche Therapie für alle SepsispatientInnen geeignet ist, sondern eher, welche Therapie für wen zum jeweiligen Zeitpunkt sinnvoll erscheint.

Darüber hinaus könnte die Intensivmedizin der Zukunft zunehmend durch datengetriebene und lernende Systeme geprägt werden. Echtzeitbiomarker, Multiomics-Analysen und KI-gestützte Verfahren zur Phänotypisierung könnten es ermöglichen, biologische Subgruppen bereits innerhalb der ersten Stunden präzise zu identifizieren. Digitale Zwillinge und prädiktive Modelle könnten individuelle Krankheitsverläufe simulieren und dadurch therapeutische Entscheidungen unterstützen. Gleichzeitig werden adaptive Plattformstudien dazu beitragen, vielversprechende Therapieansätze schneller zu evaluieren und Studienprotokolle dynamisch an neue Erkenntnisse anzupassen. Langfristig könnte sich die Sepsisbehandlung von einer einmaligen Therapieentscheidung hin zu einem Prozess der kontinuierlichen Neubewertung und Anpassung entwickeln.

## Schlussfolgerungen

Die lange Geschichte negativer Sepsisstudien sollte nicht als Beweis für das Scheitern kausaler Therapien interpretiert werden. Vielmehr spiegeln die Ergebnisse die enorme biologische Komplexität der Erkrankung und die Grenzen traditioneller Studiendesigns wider.

Die drei aktuellen positiven Studien zeigen, dass präzisionsmedizinische Konzepte, biologische Stratifizierung, adaptive Protokolle und innovative statistische Methoden neue Möglichkeiten eröffnen. Sie markieren den Übergang von einer rein syndromorientierten Betrachtung hin zu einer biologisch fundierten Charakterisierung der Erkrankung. Sepsis sollte demnach nicht länger als einheitliches Krankheitsbild verstanden werden, sondern als ein Syndrom mit sehr unterschiedlichen pathophysiologischen Zuständen. Die eigentliche Innovation der aktuellen Sepsisforschung liegt nicht in einzelnen neuen Medikamenten, sondern in einem grundlegenden Wechsel des Denkmodells: weg von der Behandlung eines Syndroms hin zur Behandlung biologisch definierter PatientInnengruppen. Ob sich dadurch erstmals wirksame kausale Therapien etablieren lassen, werden die kommenden Jahre zeigen.

## Fazit für die Praxis

- Sepsis bleibt trotz Fortschritten eine häufige Todesursache ohne spezifische kausale Therapie.
- Hauptgründe für das Scheitern früherer Studien sind die ausgeprägte biologische und klinische Heterogenität der PatientInnen sowie ungeeignete Studiendesigns und Endpunkte.
- Klassische RCTs mit breiten Populationen und Mortalität als primärem Endpunkt können relevante Therapieeffekte häufig nicht sichtbar machen.
- Präzisionsmedizinische Ansätze mit Stratifizierung zeigen erstmals konkrete klinische Vorteile.
- Die Zukunft der Sepsisforschung liegt somit in individualisierten Therapiestrategien und innovativen Studienkonzepten.
